# Potential role of dietary red onion peel polyphenol-rich extract in enhancing growth, feed utilization, and antioxidant and immune responses in Nile tilapia (*Oreochromis niloticus*) cultured at high stocking density

**DOI:** 10.1007/s10695-026-01651-x

**Published:** 2026-02-20

**Authors:** Adham A. Al-Sagheer, Ahmed M. N. Ayyat, Norhan H. Ahmed, Mohamed S. Ayyat, Ali Osman, Khaled M. Abd El-Latif

**Affiliations:** 1 Department of Animal Production, Faculty of Agriculture, Zagazig University, Zagazig, 44511 Egypt; 2 Department of Fish Nutrition and Feed Technology, Central Laboratory for Aquaculture Research, Abassa, Abu Hammad, Sharkia Egypt; 3 Biochemistry Department, Faculty of Agriculture, Zagazig University, Zagazig, 44511 Egypt; 4 Specialized Hospital, Ain Shams University, Cairo, 11588 Egypt

**Keywords:** Nile tilapia, Stocking rate, Onion extract, Feed additives, Productive performance

## Abstract

This study evaluated the impact of dietary inclusion of red onion peel extract (ROPE) on body composition, growth, feed utilization, immune response, antioxidant status, and economic efficiency of *Oreochromis niloticus* reared under high stocking density. A total of 630 mono-sex Nile tilapia (6.09 ± 0.11 g) were randomly distributed into four groups (*n* = 3 outdoor concrete tanks per treatment; 1 m^3^ water/tank). The experimental groups were: G1, low stocking density (30 fish/tank, basal diet); while G2, G3, and G4, high stocking density (60 fish/tank) supplemented with 0, 250, and 500 mg/kg ROPE, respectively. Fish were fed to apparent satiation three times daily for 14 weeks. Results showed that fish subjected to high stocking density (G2) exhibited reduced weight gain, final body weight and protein efficiency ratio, along with elevated feed conversion ratio compared to low-density group (G1). However, supplementation with ROPE significantly improved these growth and feed utilization indices (*P* < 0.01). Whole-body composition (moisture, protein, lipid, ash) did not differ significantly among treatments (*P* > 0.05). Additionally, G3 and G4 showed enhanced blood total protein and albumin levels, reduced alanine aminotransferase activity, and lower serum lipid peroxidation (*P* < 0.05). Antioxidant enzyme activities (catalase and reduced glutathione) and immune parameters (lysozyme activity and immunoglobulin M) were significantly elevated in ROPE-supplemented groups (*P* < 0.05). Economically, G4 yielded the highest final and relative profit margins among all high-density groups. In conclusion, dietary supplementation with 500 mg/kg ROPE effectively mitigated the adverse impacts of high stocking density by enhancing growth metrics, physiological health, and economic returns in Nile tilapia.

## Introduction

In global aquaculture, the tilapia industry represents one of the most resilient and rapidly expanding sectors. Among tilapia species, Nile tilapia (*Oreochromis niloticus*) stands out as the most widely cultivated and commercially significant worldwide (Sriprateep et al. 2025). To meet increasing production demands, intensive aquaculture systems frequently employ high stocking; however, excessive crowding can compromise fish health and productivity (Fuentes-Andraca et al. 2025; Seo and Park 2023). High stocking density is well known to stress fish, leading to reduced growth and survival, disrupted metabolism, altered behavior (e.g. social hierarchy), and suppressed immunity (Abdel Fattah et al. 2020; Liu et al. 2018). Biologically, crowding increases competition for resources and induces chronic stress, which diverts energy to homeostasis at the expense of growth (Diao et al. 2023). Under chronic crowding, fish show elevated oxidative stress and weakened antioxidant defenses, along with impaired immune function and higher disease susceptibility (Elashry et al. 2024; Shourbela et al. 2021). Economically, the negative effects of overcrowding can outweigh any gains in yield. Indeed, it has been noted that if stocking density is not properly optimized, profitability is reduced by chronic growth suppression or disease outbreaks (Aïzonou et al. 2023; Ayyat et al. 2024; Obiero et al. 2022). To mitigate crowding stress, nutritional strategies increasingly employ bioactive feed additives. Plant-derived phenolic compounds and natural antioxidants enhance growth, feed efficiency, and immune and antioxidant responses under stress (Almarri et al. 2023; Hu et al. 2025; Mansoori et al. 2024). Such eco-friendly supplements offer sustainable alternatives to synthetic additives, improving stress resilience and disease resistance in aquaculture.

Among natural supplements, *Allium* species (garlic and onion) have long been recognized for bioactive compounds (flavonoids, sulfur compounds) with antimicrobial and antioxidant properties (Singh et al. 2025). Studies showed that onion waste contains high levels of flavonoids, especially quercetin and its derivatives, which are potent natural antioxidants (Wojtczak et al. 2025). In aquaculture, dietary inclusion of onion (*Allium cepa*) has been shown to improve fish performance under stress conditions. For instance, dietary onion pulp powder improved growth and immunity in tilapia and common carp (Akrami et al. 2015; Saleh et al. 2015). Similarly, the combination of onion peel and pawpaw seed mixture boosted immune cell activity and antioxidant enzyme responses in *Clarias gariepinus* (Fawole et al. 2020). Moreover, Aluta et al. (2021) confirmed that onion peel-supplemented diets improved blood chemistry, feed utilization, growth indicators, and antioxidant enzyme activities in *C. gariepinus*. In tilapia, supplementation with onion extract enhanced growth, nonspecific immune responses, reduced oxidative stress, lower cadmium accumulation in tissues, upregulated expression of immune-related genes, and improved resistance against *Saprolegnia parasitica* infection (Elgendy et al. 2023). Likewise, Reda et al. (2024) reported that water treatment with onion extract significantly improved blood hematology and upregulated innate immune indicators (lysozyme, anti-proteases, nitric oxide) in tilapia infested with gill parasites. These findings highlight that onion-derived ingredients can act as immunostimulants and antioxidants in tilapia, consistent with its traditional health-related uses.

To our knowledge, no previous studies have investigated the dietary supplementation of red onion peel extract (ROPE) under high stocking density conditions. Given its documented biological activities and beneficial effects on fish growth and health, it was hypothesized that dietary inclusion of ROPE could mitigate the adverse impacts of crowding stress in Nile tilapia. Thus, the objective of this study was to evaluate the effects of two inclusion levels of ROPE (250 and 500 mg/kg diet) on the growth, feed conversion efficiency, oxidative-immunological responses, whole-body composition, and overall economic returns of *O. niloticus* cultured under high stocking density over a 14-week feeding period.

## Materials and methods

### Preparation of plant extract and experimental diets

The outer skin and non-edible layers of fresh onions (*Allium cepa* L.) were carefully removed, and 100 g of onion peels were combined with 250 mL of 70% ethanol. The mixture was homogenized thoroughly and stirred continuously for 2 h. Following extraction, the mixture was filtered through Whatman No. 2 filter paper. The ethanolic filtrate was subsequently concentrated under reduced pressure using a BüCHI water bath evaporator (Model B-480) at 45 °C. The concentrated extract was then subjected to freeze-drying using a Heto PowerDry LL 300 freeze dryer (Thermo Electron Corporation). The dried material was labeled as ROPE. The total phenolic content of the extract was quantified using the Folin–Ciocalteu colorimetric assay, following the procedure outlined by Lister and Wilson (2001), and reported as mg gallic acid equivalents (GAE)/g extract. Flavonoid content was assessed according to Yıldırım et al. (2001) and expressed as mg quercetin equivalents (QE)/g extract. The total phenolic content in the extract was found to be 388.14 ± 2.98 mg GAE/g extract, while the total flavonoid content was 227.49 ± 2.84 mg QE/g extract.

The feed ingredients were finely milled into a powder. The required amounts of ROPE (250 and 500 mg/kg diet) were first thoroughly mixed with a small portion of the basal diet. This premix was then blended with the remaining feed ingredients to achieve uniform distribution. The final mixture was moistened with water to form a homogeneous paste, then extruded through an electric meat mincer with a 2.0 mm die to produce uniform pellets. After air-drying at ambient temperature, the pellets were stored at 4 °C in sealed plastic bags. The basal diet’s formulation and proximate composition are listed in Table 1. Diet formulations were based on the nutritional requirements for tilapia outlined by NRC (2011).

**Table 1:** Formulation and proximate chemical analysis of the basal diet

Ingredients	Content **(**g/100 g diet**)**
Corn meal	33
Fish meal, 60%CP	10
Corn gluten, 60%CP	12
Soybean meal, 44%CP	31
Wheat meal	8
Sunflower oil	2
Fish oil	2
Vitamins and minerals^1^	2
Total	100
Proximate composition (%)	
Crude protein	30.15
Ether extract	7.38
Ash	5.46
Crude fiber	5.03
Nitrogen free extract^2^	51.98
Gross energy (MJ/kg diet) ^3^	18.89

^1^Vitamin and mineral mixture content per kg of diet: 30 mg Fe, 15 mg Mg, 75 mg K, 4 mg Cu, 42 mg Zn, 0.11 mg Co, 0.4 mg I, 0.04 mg Se, 1.6 mg Mn, and 0.005 mg Mo; along with 4000 IU vitamin A, 600 IU vitamin D₃, 20 mg vitamin E, 5 mg vitamin K₃, 3.6 mg vitamin B₁, 6 mg vitamin B₂, 14.4 mg vitamin B₃, 12 mg vitamin B₅, 3.5 mg vitamin B₆, 0.02 mg vitamin B₁₂, 0.9 mg folic acid, 0.07 mg biotin, 50 mg vitamin C, and 300 mg inositol

^2^Nitrogen-free extract (%) was calculated as: organic matter (%) – [protein (%) + ether extract (%) + crude fiber (%)]

^3^Gross energy was calculated using the caloric values: crude protein (23.4 kJ/g), ether extract (39.2 kJ/g), and nitrogen free extract (17.2 kJ/g) (NRC 2011)

### Identification of chemical components of ROPE by GC–MS

The chemical constituents of ROPE were identified using gas chromatography–mass spectrometry (GC–MS) on a GC–TSQ mass spectrometer (Thermo Scientific, Austin, TX, USA) equipped with a TG–5MS capillary column (30 m × 0.25 mm × 0.25 µm film thickness). The oven temperature was initially set at 60 °C, then gradually increased at a rate of 5 °C per minute up to 250 °C, held for 2 min, and subsequently ramped to 300 °C at 30 °C per minute. The injector temperature was maintained at 270 °C. Helium served as the carrier gas at a constant flow rate of 1 mL/min. A 1 µL aliquot of each diluted sample was automatically injected using an AS3000 autosampler operating in split mode, with a solvent delay of 4 min. Electron ionization (EI) was performed at 70 eV, scanning a mass range of *m/z* 50–650 in full-scan mode. The ion source and transfer line temperatures were set to 200 °C and 280 °C, respectively. Compound identification was achieved by matching the obtained mass spectra with those in the NIST14 and WILEY09 spectral libraries.

### Fish, treatments, and culture conditions

The study was conducted at the Central Laboratory for Aquaculture Research, Abbassa, Abu Hammad, Sharkia, Egypt. Mono-sex juvenile male *O. niloticus* were obtained from a commercial hatchery in Sharkia Governorate, Egypt. Upon arrival, fish were acclimated for 10 days to the experimental conditions in outdoor concrete tanks (1.0 × 1.5 × 1.0 m). Each tank was filled with a total water volume of 1 m^3^. Continuous aeration was supplied using air blowers connected to diffusers installed in each concrete tank. A continuous water flow of approximately 5 L/min was maintained to ensure adequate oxygenation and water quality. In addition, about one-third of the tank water was manually replaced weekly with dechlorinated freshwater during routine cleaning to remove settled waste and maintain optimal hygiene.

Fish were fed the formulated control diet (containing 30.15% crude protein and 7.38% lipid) during the acclimation phase. After acclimation, 630 fish (6.09 ± 0.11 g) were randomly divided among four treatment groups, with three tanks each. The experimental groups were as follows: a low-density control group (G1, 30 fish/m^3^, basal diet), a high-density control group (G2, 60 fish/m^3^, basal diet), and two high-density groups (G3 and G4, 60 fish/m^3^) fed diets supplemented with 250 mg/kg and 500 mg/kg of ROPE, respectively. The low-density group (G1) served as a baseline control and was used for comparison with the high-density control group (G2) to evaluate the effect of stocking density without additives. The high-density groups supplemented with ROPE (G3 and G4) were compared with the high-density control group (G2) to assess the potential effects of dietary supplementation under crowding conditions. The selected stocking densities were based on (Ayyat et al. 2024), who reported that the higher density (60 fish/m^3^) induced impaired growth and biochemical performance in Nile tilapia compared with the lower density (30 fish/m^3^). Fish were hand-fed to apparent satiation three times daily at 10:00, 13:00, and 17:00. Throughout the 14-week feeding period, water quality was regularly monitored and maintained at levels appropriate for Nile tilapia culture. The average recorded values were: pH 7.49 ± 0.09, dissolved oxygen 5.47 ± 0.17 mg/L, temperature 27.00 ± 0.63 °C, nitrite 0.15 ± 0.03 mg/L, and nitrate 2.33 ± 0.23 mg/L. Temperature and dissolved oxygen were measured using a portable oxygen and temperature meter (HI9146, Hanna Instruments, Romania), while pH, nitrate, and nitrite concentrations were determined with a multiparameter bench meter (HI83205, Hanna Instruments, Romania). Water quality remained consistent across all treatment groups, with no significant differences detected. The fish were exposed to a natural photoperiod of roughly 14 h of light and 10 h of darkness.

### Growth and feed utilization indices

Each tank was considered an independent experimental unit, and all growth and feed utilization data were calculated based on the tank level. The following equations were used to assess growth performance and feed utilization efficiency:

1. Daily gain (DG, g/day) = (FBW − IBW)/D;
2. Feed conversion ratio (FCR) = FI/(FBW − IBW);
3. Daily protein intake (DPI, g/day) = (FI × CP)/D;
4. Protein efficiency (PE)/day = DG/DPI;
5. Relative growth rate (RGR, %) = 100 × [(FBW − IBW)/IBW];
6. Survival rate (%) = (FN/IN) × 100.

Where: FBW = Final body weight (g); IBW = Initial body weight (g); D = Duration of the experiment (days); FI = Feed intake (g); CP = Crude protein content of the diet (g/g); IN = Initial number of fish; FN = Final number of fish.

### Proximate body composition analysis

Following the feeding trial, five fish per tank (15 per treatment) were randomly selected and stored at –20 °C for whole-body proximate composition assessment. Moisture content was determined by drying the samples in an oven at 105 °C until a constant weight was achieved. Analyses followed AOAC (2012) standard protocols: crude protein was assessed by Kjeldahl digestion (N × 6.25); crude fat was extracted from dried sample using a Soxhlet apparatus with petroleum ether (60–80 °C); and ash content was obtained via 6-h incineration at 550 °C.

### Blood sampling

Following the 14-week feeding trial, fish were fasted for 24 h prior to blood collection. Five fish were randomly selected from each replicate (15 fish per treatment group) and anesthetized using a clove oil solution at a concentration of 200 mg/L (Hassan et al. 2021). Using 1 ml plastic syringes without anticoagulant, blood was drawn from the caudal vessels and left to clot at 4 °C. After clotting, blood samples were centrifuged (1500 × g, 15 min) to obtain serum, which was stored at –20 °C for later biochemical and immunological evaluation.

### Serum protein profile and renal-liver function evaluation

The serum protein profile, including total protein (TP) and albumin (ALB) (Sundeman 1964), was measured using commercial colorimetric kits (Biodiagnostic, Giza, Egypt; catalog numbers TP 20 20 and AB 10 10, respectively). The level of globulin (GLB) was determined by the difference between TP and ALB. Aspartate aminotransferase (AST) and alanine aminotransferase (ALT), key liver function enzymes, were assessed spectrophotometrically using specific Biodiagnostic kits (catalog numbers AS 10 61 and AL 10 31, respectively) according to Reitman and Frankel (1957). Additionally, serum levels of urea (Patton and Crouch 1977) and creatinine (Larsen 1972) were determined using commercial colorimetric kits from the same supplier (catalog numbers UR 21 10 and CR 12 50, respectively).

### Antioxidant and humoral immune parameter analysis

Antioxidant biomarkers, including glutathione (GSH), catalase (CAT), and malondialdehyde (MDA), a key indicator of lipid peroxidation, were quantified using commercially available assay kits (BioSource Inc., San Diego, CA, USA). Serum levels of immunoglobulin M (IgM) were quantified using a commercial enzyme-linked immunosorbent assay (ELISA) kit (MyBioSource, San Diego, USA; Catalogue No. MBS042355). The assay was performed strictly following the manufacturer's instructions. Absorbance values were measured using a microplate reader, and IgM concentrations were calculated based on the standard curve provided with the kit. Lysozyme activity in the serum was determined spectrophotometrically according to the method described by Ellis (1990), with modifications based on Ghareghanipoora et al. (2014). The assay measured the rate of lysis of *Micrococcus lysodeikticus* (Sigma Co., USA) as the substrate. Briefly, serum samples were incubated with a suspension of freeze-dried *M. lysodeikticus*, and the reduction in optical density at 540 nm was recorded over a 5-min period using a 5010 photometer (BM Co., Germany). A standard curve was established using serial dilutions of lyophilized chicken egg-white lysozyme (Sigma Co., USA), allowing lysozyme activity in the serum samples to be expressed in equivalent units.

### Economic evaluation

Economic performance was evaluated according to Ayyat et al. (2018). Final margin was calculated by subtracting feed costs from the total revenue, based on a market price of US $2.24 per kg of fish. Final margin (USD) = [Total biomass (kg) × 2.24 US $] – [Feed intake (kg) × feed cost]. Relative margin (%) was derived by adjusting net profit according to the survival rate: Relative margin = Final margin × (Survival rate/100). All other operational costs were considered equal among treatments.

### Data analysis

The tank was considered the experimental unit for growth and feed utilization data. Data were checked for normality (Shapiro–Wilk test) and homogeneity of variances (Levene’s test) prior to analysis. A one-way analysis of variance (ANOVA) was performed on all data to assess the effect of dietary onion extract on all measured parameters. Tukey’s HSD test was performed for pairwise comparisons when ANOVA indicated significant differences (*p* < 0.05). All statistical analyses were conducted using SAS software (SAS Institute Inc., Cary, NC, USA; version 9.0, 2002).

## Results

### GC–MS identification of chemical components of ROPE

**Fig. 1:**
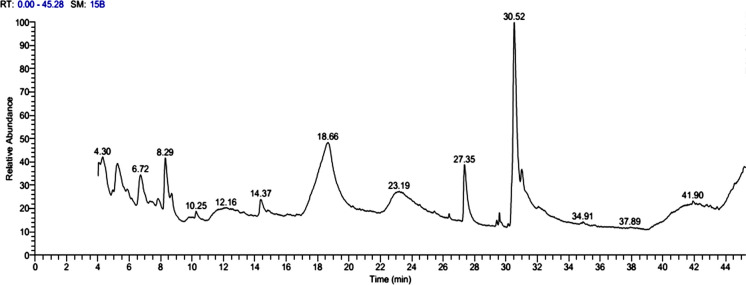
Gas chromatogram of the red onion peel polyphenol-rich extract (ROPE) obtained by gas chromatography-mass spectrometry (GC–MS). The chromatogram shows the relative abundance of detected compounds over a retention time range of 0–45 min

Gas chromatographic analysis of ROPE identified 22 bioactive compounds (Table 2; Fig. 1). Oleic acid was the major constituent (35.17%), followed by guanosine (15.37%), hexadecanoic acid (8.55%), and 2,3-dihydro-3,5-dihydroxy-6-methyl-4H-pyran-4-one (DDMP; 7.57%). Other notable compounds included furaneol (6.75%), 2-deoxy-2-fluoro-1,6-anhydro-α-D-glucopyranose (6.55%), and 2-hydroxy-3-pentanone (6.24%). Several minor constituents (< 3%) such as Z-8-methyl-9-tetradecenoic acid, 2-aminoethanethiol hydrogen sulfate (ester), 5-methyl-2-ethylamino-2-thiazoline, and dodecanoic acid, 3-hydroxy-, were also detected.

**Table 2:** Retention time and peak area (%) of the different compounds found in red onion peel polyphenol-rich extract analyzed by GC–MS

	Compound Name	MF	MW	RT (min)	Area %
1	Oleic acid	C_18_H_34_O_2_	282	30.52	35.17
2	Guanosine (2-amino-9-(β-D-ribofuranosyl)−3,9-dihydro-purin-6-one)	C_10_H_13_N_5_O_5_	283	18.58	15.37
3	Hexadecanoic acid	C_16_H_32_O_2_	256	27.35	8.55
4	2,3-dihydro-3,5-dihydroxy-6-methyl-4H-pyran-4-one (DDMP)	C_6_H_8_O_4_	144	8.29	7.57
5	Furaneol	C_6_H_8_O_3_	128	6.71	6.75
6	2-Deoxy-2-fluoro-1,6-anhydro-α-d-glucopyranose	C_8_H_16_N_2_O_7_	252	4.31	6.55
7	2-Hydroxy-3-pentanone	C_5_H_10_O_2_	102	5.24	6.24
8	Z-8-Methyl-9-tetradecenoic acid	C_15_H_28_O_2_	240	14.36	2.63
9	2-Aminoethanethiol hydrogen sulfate (ester)	C_2_H_7_NO_3_S_2_	157	23.32	1.24
10	5-Methyl-2-ethylamino-2-thiazoline	C_6_H_12_N_2_S	144	8.71	1.10
11	Dodecanoic acid, 3-hydroxy-	C_12_H_24_O_3_	216	29.57	1.05
12	Desulphosinigrin	C_10_H_17_NO_6_S	279	23.19	0.98
13	3-O-Hexopyranosylhex-2-ulofuranosyl hexopyranoside	C_18_H_32_O_16_	504	7.86	0.97
14	2-Aminoethanethiol hydrogen sulfate (ester)	C_2_H_7_NO_3_S_2_	157	23.43	0.96
15	2(3H)-Furanone, 5-heptyldihydro-	C_11_H_20_O_2_	184	10.25	0.95
16	6-Nonenoic acid, methyl ester	C_10_H_18_O_2_	170	4.06	0.87
17	Acetic acid, nonyl ester	C_11_H_22_O_2_	186	7.81	0.66
18	9-Hexadecenoic acid	C_16_H_30_O_2_	254	11.61	0.65
19	7-Hexadecenoic acid, methyl ester, (Z)-	C_17_H_32_O_2_	268	4.10	0.58
20	E-8-Methyl-7-dodecen-1-ol acetate	C_15_H_28_O_2_	240	11.69	0.50
21	Oxiraneundecanoic acid, 3-pentyl-, methyl ester	C_19_H_36_O_3_	312	26.37	0.36
22	Desulphosinigrin	C_10_H_17_NO_6_S	279	23.27	0.31
	**Total**				**100**

*RT* retention time, *MF* Molecular Formula, *MW* Molecular weight

### Growth performance and survival rate

Growth performance and survival data are summarized in Table 3. Regardless of dietary supplementation, FBW and weight gain during weeks 0–14 were significantly reduced in fish reared at high stocking density without ROPE (G2) compared to low stocking density (G1) (*P* < 0.05). Supplementation with ROPE at both 250 and 500 mg/kg under high stocking density (G3 and G4) significantly improved body weight, DG, and RGR at most time intervals compared to the unsupplemented high-density group (G2; *P* < 0.05). However, RGR during weeks 0–6 and 6–14 showed no significant differences among groups (*P* > 0.05). Survival rate did not differ significantly among groups (Table 3).

**Table 3:** Effects of dietary red onion peel extract (ROPE) on growth performance and survival of Nile tilapia reared under high stocking density

Items	Experimental groups^1^				*P*-value
	G1	G2	G3	G4	
Body weight (g/fish)					
Week 0	6.06 ± 0.06	6.06 ± 0.05	6.14 ± 0.07	6.11 ± 0.07	0.752
Week 6	40.18 ± 0.10^b^	39.94 ± 0.06^b^	41.01 ± 0.30^a^	41.45 ± 0.15^a^	0.001
Week 14	56.19 ± 0.36^b^	53.58 ± 0.30^c^	60.14 ± 1.20^a^	61.17 ± 0.56^a^	< 0.001
Daily gain (g/day)					
Weeks 0–6	0.813 ± 0.003^b^	0.807 ± 0.003^b^	0.830 ± 0.007^a^	0.841 ± 0.005^a^	0.003
Weeks 6–14	0.286 ± 0.008^b^	0.244 ± 0.005^b^	0.342 ± 0.026^a^	0.352 ± 0.013^a^	0.003
Weeks 0–14	0.512 ± 0.004^b^	0.485 ± 0.004^c^	0.551 ± 0.013^a^	0.562 ± 0.005^a^	< 0.001
Relative growth rate (%)					
Weeks 0–6	563.55 ± 6.33	559.74 ± 6.92	568.17 ± 7.90	578.58 ± 10.12	0.442
Weeks 6–14	39.87 ± 1.15	34.13 ± 0.67	46.71 ± 3.92	47.57 ± 1.89	0.010
Weeks 0–14	827.98 ± 4.95^b^	784.95 ± 11.56^b^	880.24 ± 28.55^a^	901.00 ± 5.37^a^	0.003
Survival rate (%)					
Weeks 0–14	93.33 ± 0.00	93.33 ± 0.96	95.00 ± 0.96	95.56 ± 0.56	0.140

Values are means ± SEM. Different superscript letters within the same row indicate significant differences (*P* < 0.05). ^1^ G1: Low stocking density (30 fish/m^3^) without ROPE supplementation; G2: High stocking density (60 fish/m^3^) without ROPE; G3: High stocking density (60 fish/m^3^) with 250 mg/kg dietary ROPE; G4: High stocking density (60 fish/m^3^) with 500 mg/kg dietary ROPE

### Feed intake and feed efficiency

Table 4 indicates that both daily feed intake (DFI) and daily protein intake (DPI) increased significantly in fish reared under high stocking density (G2) compared to low density (G1) during weeks 0–6 and 0–14 (*P* < 0.001). Additionally, FCR and PER were significantly impaired in G2 across all time intervals (*P* < 0.05). Dietary supplementation with ROPE had a significant effect on feed intake and utilization under high stocking density. During weeks 0–6 and over the entire 0–14-week period, DFI increased significantly in the ROPE-supplemented groups (G3 and G4) (*P* < 0.001). FCR was significantly improved in both supplemented groups throughout all experimental periods (*P* < 0.01), while PER was significantly higher during weeks 6–14 and over the entire trial in G3 and G4 compared to G2 (*P* < 0.01).

**Table 4:** Effects of dietary red onion peel extract (ROPE) on feed efficiency of Nile tilapia reared under high stocking density

Items	Experimental groups^1^				*P*-value
	G1	G2	G3	G4	
Daily feed intake (g/day)					
Weeks 0–6	0.512 ± 0.001^c^	0.811 ± 0.001^b^	0.813 ± 0.001^ab^	0.814 ± 0.001^a^	< 0.001
Weeks 6–14	1.392 ± 0.003	1.401 ± 0.016	1.401 ± 0.001	1.404 ± 0.001	0.777
Weeks 0–14	1.015 ± 0.002^b^	1.148 ± 0.009^a^	1.149 ± 0.001^a^	1.151 ± 0.000^a^	< 0.001
Feed conversion ratio (g feed: g gain)					
Weeks 0–6	0.63 ± 0.01^c^	1.01 ± 0.01^a^	0.98 ± 0.01^b^	0.97 ± 0.01^b^	< 0.001
Weeks 6–14	4.94 ± 0.16^b^	5.72 ± 0.21^a^	4.37 ± 0.37^c^	4.23 ± 0.15^c^	0.002
Weeks 0–14	2.00 ± 0.02^ab^	2.09 ± 0.04^a^	1.94 ± 0.05^b^	1.91 ± 0.02^b^	< 0.001
Daily protein intake (g/day)					
Weeks 0–6	0.154 ± 0.0001^b^	0.245 ± 0.0002^a^	0.245 ± 0.0001^a^	0.245 ± 0.0001^a^	< 0.001
Weeks 6–14	0.420 ± 0.001	0.422 ± 0.005	0.423 ± 0.0003	0.423 ± 0.000	0.750
Weeks 0–14	0.306 ± 0.0006^b^	0.346 ± 0.003^a^	0.346 ± 0.0003^a^	0.347 ± 0.000^a^	< 0.001
Protein efficiency					
Weeks 0–6	5.260 ± 0.022 ^a^	3.298 ± 0.013 ^c^	3.387 ± 0.030 ^b^	3.429 ± 0.020 ^b^	< 0.001
Weeks 6–14	0.682 ± 0.019 ^b^	0.577 ± 0.019 ^c^	0.809 ± 0.063 ^a^	0.832 ± 0.030 ^a^	0.004
Weeks 0–14	1.672 ± 0.012 ^a^	1.401 ± 0.021 ^c^	1.591 ± 0.037 ^b^	1.619 ± 0.014 ^b^	< 0.001

Values are means ± SEM. Different superscript letters within the same row indicate significant differences (*P* < 0.05). ^1^ G1: Low stocking density (30 fish/m^3^) without ROPE supplementation; G2: High stocking density (60 fish/m^3^) without ROPE; G3: High stocking density (60 fish/m^3^) with 250 mg/kg dietary ROPE; G4: High stocking density (60 fish/m^3^) with 500 mg/kg dietary ROPE

### Chemical body composition

Table 5 presents the results of whole-body chemical composition of Nile tilapia reared under different stocking densities and dietary ROPE supplementation levels. Whole-body moisture, crude protein, crude lipid, and ash contents were not significantly affected by stocking density or dietary ROPE supplementation (*P* > 0.05).

**Table 5:** Effects of dietary red onion peel extract (ROPE) on whole-body chemical composition (% on dry matter basis) of Nile tilapia reared under high stocking density

Items	Experimental groups^1^				*P*-value
	G1	G2	G3	G4	
Moisture	71.64 ± 0.36	72.61 ± 0.69	70.94 ± 0.29	71.46 ± 0.10	0.112
Crude protein	57.63 ± 0.34	57.08 ± 0.22	57.98 ± 0.20	57.81 ± 0.37	0.224
Crude lipids	20.19 ± 0.44	21.80 ± 0.79	20.62 ± 0.28	20.82 ± 0.23	0.199
Ash	20.81 ± 0.36	20.12 ± 0.29	20.94 ± 0.44	20.75 ± 0.20	0.374

Values are means ± SEM.^1^ G1: Low stocking density (30 fish/m^3^) without ROPE supplementation; G2: High stocking density (60 fish/m^3^) without ROPE; G3: High stocking density (60 fish/m^3^) with 250 mg/kg dietary ROPE; G4: High stocking density (60 fish/m^3^) with 500 mg/kg dietary ROPE

### Hepatic and renal biochemical parameters

Table 6 demonstrates that high stocking density (G2) significantly reduced serum TP and ALB levels and increased ALT activity compared to fish reared at low density (G1) (*P* < 0.05). No significant differences were observed between G1 and G2 for GLB, AST, creatinine, or urea levels (*P* > 0.05). Dietary supplementation with ROPE significantly improved blood biochemical responses in fish reared under high stocking density. Both 250 mg/kg (G3) and 500 mg/kg (G4) significantly increased TP and ALB concentrations compared to the unsupplemented high-density group (G2) (*P* < 0.05). Additionally, ALT activity was significantly reduced in G3 and G4 relative to G2 (*P* < 0.05). Creatinine, GLB, AST, and urea levels remained unaffected by ROPE supplementation (*P* > 0.05).

**Table 6:** Effects of dietary red onion peel extract (ROPE) on blood biochemical parameters of Nile tilapia reared under high stocking density

Items	Experimental groups^1^				*P*-value
	G1	G2	G3	G4	
Total protein(g/dL)	5.15 ± 0.10^a^	4.71 ± 0.10^b^	5.34 ± 0.09^a^	5.42 ± 0.11^a^	0.005
Albumin (g/dL)	3.14 ± 0.07^a^	2.63 ± 0.05^b^	3.38 ± 0.28^a^	3.44 ± 0.13^a^	0.026
Globulin (g/dL)	2.01 ± 0.12	2.08 ± 0.14	1.96 ± 0.20	1.98 ± 0.09	0.937
ALT (U/L)	19.28 ± 0.04^b^	21.70 ± 0.67^a^	19.90 ± 0.18^b^	19.80 ± 0.28^b^	0.009
AST (U/L)	23.34 ± 0.33	24.79 ± 0.28	23.22 ± 0.71	23.37 ± 0.12	0.088
Creatinine (mg/dL)	0.90 ± 0.05	1.05 ± 0.05	0.91 ± 0.01	0.89 ± 0.12	0.351
Urea (mg/dL)	12.89 ± 0.33	13.75 ± 0.37	12.89 ± 0.40	12.81 ± 0.31	0.266

Values are means ± SEM. Different superscript letters within the same row indicate significant differences (*P* < 0.05). ^1^ G1: Low stocking density (30 fish/m^3^) without ROPE supplementation; G2: High stocking density (60 fish/m^3^) without ROPE; G3: High stocking density (60 fish/m^3^) with 250 mg/kg dietary ROPE; G4: High stocking density (60 fish/m^3^) with 500 mg/kg dietary ROPE

### Antioxidant and immune responses

**Fig. 2:**
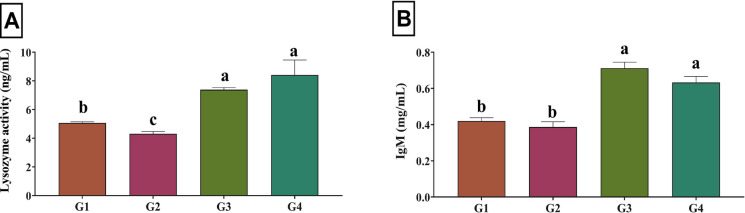
Serum levels of (**A**) lysozyme (*P* = 0.008) and (**B**) immunoglobulin M (*P* = 0.001) in Nile tilapia reared under high stocking density, as influenced by dietary supplementation with red onion peel phenolic extract (ROPE). Values are means ± SEM. Different superscript letters within the same column indicate significant differences (*P* < 0.05). G1: Low stocking density (30 fish/m^3^) without ROPE; G2: High stocking density (60 fish/m^3^) without ROPE; G3: High stocking density (60 fish/m^3^) with 250 mg/kg dietary ROPE; G4: High stocking density (60 fish/m^3^) with 500 mg/kg dietary ROPE

**Fig. 3:**
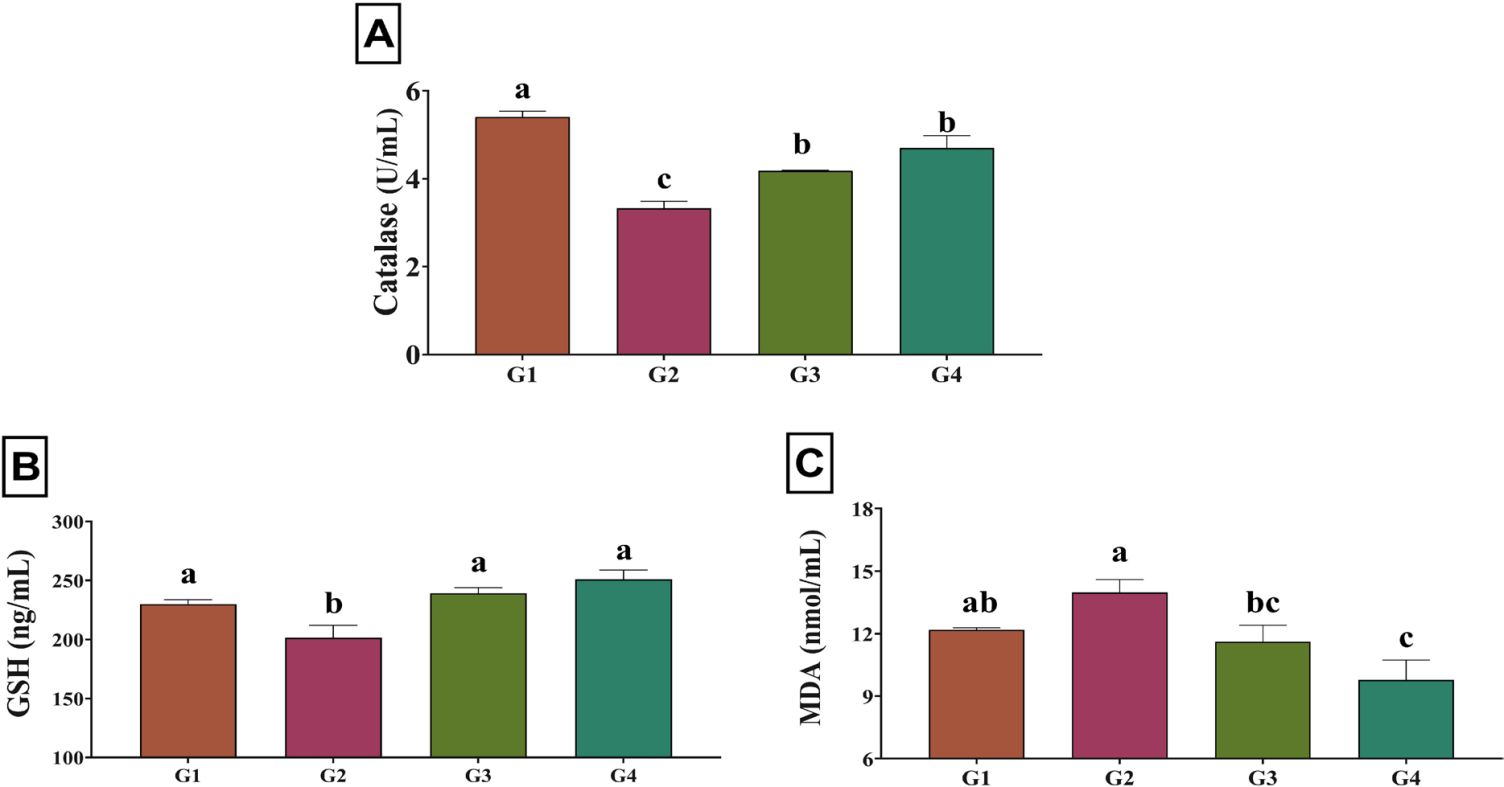
Serum levels of (**A**) catalase (*P* < 0.001), (**B**) reduced glutathione (GSH; *P* = 0.007), and (**C**) malondialdehyde (MDA; *P* = 0.009) in Nile tilapia reared under high stocking density, as affected by dietary supplementation with red onion peel extract (ROPE). Values are means ± SEM. Different superscript letters within the same column indicate significant differences (*P* < 0.05). G1: Low stocking density (30 fish/m^3^) without ROPE; G2: High stocking density (60 fish/m^3^) without ROPE; G3: High stocking density (60 fish/m^3^) with 250 mg/kg dietary ROPE; G4: High stocking density (60 fish/m^3^) with 500 mg/kg dietary ROPE

As shown in Fig. 2, high stocking density (G2) significantly reduced lysozyme activity and IgM levels compared to the low-density group (G1) (*P* < 0.05). Dietary ROPE supplementation significantly enhanced both parameters (*P* < 0.05). Figure 3 shows that high stocking density significantly increased MDA levels and reduced CAT and GSH activities in G2 (*P* < 0.05). ROPE supplementation (G3 and G4) significantly increased CAT and GSH activities and reduced MDA concentrations compared to G2 (*P* < 0.05), with G4 showing the most favorable antioxidant profile.

### Economic performance

The economic outcomes of Nile tilapia production under high stocking density, influenced by dietary ROPE inclusion, are shown in Table 7. Fish in the high-density, non-supplemented group (G2) showed lower final margin, return from gain, and relative margin per fish compared to the low-density control (G1). However, dietary inclusion of ROPE at both 250 mg/kg (G3) and 500 mg/kg (G4) improved all economic indicators (*P* < 0.05). The highest return and margins were recorded in G4, followed closely by G3.

**Table 7:** Effects of dietary red onion peel extract (ROPE) on economic viability of Nile tilapia reared under high stocking density

Items	Experimental groups^1^				*P*-value
	G1	G2	G3	G4	
Return from gain (US$/fish)	0.112 ± 0.001^a^	0.106 ± 0.001^b^	0.121 ± 0.002^a^	0.123 ± 0.001^a^	< 0.001
Final margin (US$/fish)	0.0359 ± 0.001^a^	0.0200 ± 0.001^c^	0.0330 ± 0.003^b^	0.0354 ± 0.001^a^	0.001
Relative margin (US$/fish)	0.0335 ± 0.001^a^	0.0187 ± 0.001^c^	0.0311 ± 0.002^b^	0.0334 ± 0.001^a^	0.001

Values are means ± SEM. Different superscript letters within the same row indicate significant differences (*P* < 0.05). ^1^ G1: Low stocking density (30 fish/m^3^) without ROPE supplementation; G2: High stocking density (60 fish/m^3^) without ROPE; G3: High stocking density (60 fish/m^3^) with 250 mg/kg dietary ROPE; G4: High stocking density (60 fish/m^3^) with 500 mg/kg dietary ROPE

## Discussion

The use of phyto-additives in aquaculture has gained considerable attention due to their potential as safer and more sustainable alternatives to conventional growth promoters and antibiotics (El-Houseiny et al. 2024; Kamble et al. 2024). The present study demonstrated that *O. niloticus* reared at high stocking density (60 fish/m^3^) without ROPE supplementation showed significantly impaired growth performance (FBW and weight gain) and nutrient conversion (FCR and PER) compared to fish at low density (30 fish/m^3^). These findings align with numerous reports that high stocking density imposes chronic stress on tilapia, reducing growth and feed conversion and altering metabolic homeostasis (Aly et al. 2025; Ayyat et al. 2024; Metwaly et al. 2025). Under high-density conditions, fish expended greater energy on continuous swimming and maintaining spatial position within the confined environment, thereby reducing the energy available for growth (Mwaura et al. 2023). The observed decline in PER and higher FCR in the G2 group suggest impaired nutrient utilization under chronic crowding stress, consistent with previous reports (Fuentes-Andraca et al. 2025). Such inefficiency may result from the diversion of metabolic energy from growth toward stress adaptation processes (Ellis et al. 2002). Moreover, increased competition for feed in densely stocked tanks can cause uneven feed distribution and heightened activity, further increasing energy expenditure and reducing feed use efficiency (Mohamed et al. 2025). Conversely, dietary ROPE at both 250 and 500 mg/kg markedly mitigated these adverse effects. This growth-promoting effect is consistent with previous studies showing that *Allium cepa*-derived additives enhance fish growth under stress: for example, *O. niloticus* fed diets containing onion showed improved weight gain and specific growth rate and elevated innate immune markers (Elgendy et al. 2023). Similarly, inclusion of onion peel powder in *Clarias gariepinus* diets significantly boosted nutrient utilization and weight gain (Aluta et al. 2021). The growth-promoting effect of ROPE is primarily attributed to its rich content of antioxidant flavonoids (e.g., quercetin) and organosulfur compounds, which confer multiple physiological benefits (Stoica et al. 2023; Zheng et al. 2023a). These bioactives mitigate oxidative stress and enhance gastrointestinal function by stimulating digestive enzyme activity and accelerating intestinal transit, leading to improved nutrient absorption and feed conversion efficiency (Platel and Srinivasan 2001). Quercetin and related polyphenols directly scavenge reactive oxygen species and inhibit pro-oxidant enzymes, suppressing ROS accumulation (Umer et al. 2023). Additionally, the prebiotic components of onion, particularly fructooligosaccharides and inulin, support the growth of beneficial gut microbiota such as *Lactobacillus* and *Bifidobacterium* spp., further contributing to intestinal health and overall physiological performance (Akrami et al. 2015).

Regarding the whole-body composition, fish reared at high stocking density (G2) showed no significant differences compared to those reared at low density. This finding is consistent with earlier reports indicating that stocking density has minimal impact on the proximate composition of tilapia, including moisture, crude protein, and mineral contents (El-Sayed 2002; Tavares et al. 2025). Similarly, fish fed ROPE-supplemented diets showed no significant differences in body composition compared to unsupplemented fish reared under high stocking density. Comparable outcomes have also been reported in fish receiving phytogenic extracts (Liu et al. 2022; Poolsawat et al. 2022).

The liver and kidneys are essential for maintaining metabolic and physiological homeostasis (El-Houseiny et al. 2025). The liver regulates carbohydrate, protein, and lipid metabolism, while the kidneys control electrolyte balance, osmoregulation, and acid–base homeostasis (John and Pasha 2024; Tao et al. 2024). Herein, fish in G2 had significantly lower serum TP and ALB, and higher ALT activity compared to low-density control, indicating impaired hepatic protein synthesis and mild liver stress under crowding. These results are consistent with reports that high density disrupts serum protein levels and causes physiological stress in tilapia (Ayyat et al. 2022, 2024). In contrast, dietary ROPE supplementation effectively mitigated these adverse effects: both the 250 and 500 mg/kg ROPE groups (G3 and G4) demonstrated significantly restored serum TP and ALB levels, along with reduced ALT activity compared to the high-density control (G2). Comparable hepatoprotective effects of phytogenic additives have been widely reported in aquaculture species. For example, Almarri et al. (2023) found that Nile tilapia fed a phenolic-rich *Annona squamosa* leaf extract exhibited enhanced hepatic and renal function when exposed to thermal stress. In another study, Akrami et al. (2015) observed marked elevations in serum TP, ALB, and GLB in juvenile beluga sturgeon receiving onion powder-supplemented diets. Additionally, Younes et al. (2021) reported a notable decrease in serum ALT and AST activities, along with a significant increase in TP, ALB, and GLB levels in *O. niloticus* fed diets enriched with *Allium cepa*, further supporting the hepatoprotective role of onion-derived compounds in maintaining liver function and overall physiological health. Onion-derived quercetin and sulfur compounds directly protect hepatocytes by scavenging ROS and inhibiting inflammatory cascades, thereby stabilizing cell membranes and reducing ALT/AST leakage (Umer et al. 2023).

High stocking density in fish is a well-documented inducer of oxidative stress, primarily through disruption of the redox balance between oxidative stress and antioxidant protection (Sahin et al. 2014; Zheng et al. 2023b). This redox imbalance facilitates excessive ROS accumulation, which in turn promotes lipid peroxidation and oxidative cellular damage (Aksu et al. 2016). In this study, chronic high stocking density impaired immune and antioxidant responses in fish, as evidenced by reduced lysozyme activity and decreased activities of key antioxidant enzymes (GSH and CAT). These patterns indicate oxidative stress and immunosuppression under high stocking density, as has been widely documented in tilapia and other aquaculture species (Aksu et al. 2016; Ayyat et al. 2024; Dawood et al. 2019; Yarahmadi et al. 2016). In fact, onion flavonoids are reported to inhibit NF-κB and suppress pro-inflammatory cytokine production (e.g., IL-1β, IL-6, TNF-α) while upregulating regulatory cytokines such as TGF-β (Kim et al. 2022; Younes et al. 2021). Notably, ROPE supplementation significantly improved immune and antioxidant responses in Nile tilapia, as evidenced by increased lysozyme activity and IgM levels, along with reduced MDA concentrations and enhanced CAT and GSH activities. These outcomes are consistent with previous studies demonstrating the immunostimulatory and antioxidant potential of *Allium cepa*. For example, Elgendy et al. (2023) reported elevated lysozyme activity and reduced oxidative markers in tilapia fed *A. cepa*-enriched diets under challenge conditions. Similarly, Aluta et al. (2021) found that dietary onion peel improved hepatic CAT and GSH activities while maintaining or reducing MDA levels in African catfish.

The observed antioxidant effects of ROPE can be attributed to its high phenolic (388 mg GAE/g) and flavonoid (227 mg QE/g) content. These bioactive compounds are well-known for their ability to scavenge ROS and terminate free radical chain reactions (Fang et al. 2002; Rice-Evans 2001). In addition, the presence of sulfur-containing compounds such as cysteine sulfoxide (CSO) and S-propenyl-CSO in onion peels (Ostrowska et al. 2004) likely contributes to ROPE’s redox-modulating activity. Sulfur is an integral component of several antioxidant molecules and enzymes, playing a critical role in enhancing cellular defense against reactive oxygen and nitrogen species (Rezk et al. 2004). Together, these phytochemicals synergistically support oxidative balance and immune competence in fish under stress conditions.

High stocking density without ROPE supplementation (G2) significantly reduced economic performance, evidenced by lower returns and profit margins compared to the low-density group (G1), due to poor growth and feed efficiency. ROPE supplementation (G3 and G4) improved economic outcomes, with the 500 mg/kg group (G4) yielding the highest net returns and margins. These improvements reflect enhanced growth and feed conversion, which increased biomass production and reduced feed cost per unit gain. Similar economic benefits of phytogenic additives under intensive aquaculture conditions have been previously documented (Hassan et al. 2025a, 2025b).

## Conclusion

This study demonstrated that dietary supplementation with ROPE effectively mitigated the adverse effects of high stocking density in Nile tilapia. Both tested doses (250 and 500 mg/kg) improved growth performance, feed efficiency, immune responses, antioxidant status, and physiological health markers. However, supplementation at 500 mg/kg yielded the best economic returns and is therefore recommended for practical application. Further research is needed to explore the underlying molecular mechanisms of ROPE’s action. Additionally, evaluating its efficacy in other fish species and under different stressors such as hypoxia or water alkalinity would help validate its broader application in aquaculture.

## Data Availability

The datasets used and/or analyzed during the current study are available from the corresponding author upon reasonable request.
